# IgE epitopes of Ara h 9, Jug r 3, and Pru p 3 in peanut-allergic individuals from Spain and the US

**DOI:** 10.3389/falgy.2022.1090114

**Published:** 2023-01-09

**Authors:** Christina M. Kronfel, Hsiaopo Cheng, Jane K. McBride, Jacqueline B. Nesbit, Rebecca Krouse, Preston Burns, Beatriz Cabanillas, Jesus F. Crespo, Robert Ryan, Reyna J. Simon, Soheila J. Maleki, Barry K. Hurlburt

**Affiliations:** ^1^ United States Department of Agriculture, Agriculture Research Service, Southern Regional Research Center, New Orleans, LA, United States; ^2^ Rho Federal Systems Division, Durham, NC, United States; ^3^ Department of Allergy, Research Institute Hospital 12 de Octubre, Madrid, Spain; ^4^ Aimmune Therapeutics, a Nestlé Health Science Company, Brisbane, CA, United States

**Keywords:** allergy diagnosis section manuscript type: original research non-specific lipid transfer proteins, Ara h 9, Jug r 3, Pru p 3, epitope, immunoglobulin E, peanut allergy, allergen

## Abstract

Non-specific lipid transfer proteins (LTPs) are well studied allergens that can lead to severe reactions, but often cause oral allergy syndrome in the Mediterranean area and other European countries. However, studies focused on LTP reactivity in allergic individuals from the United States are lacking because they are not considered major allergens. The goal of this study is to determine if differences in immunoglobulin (Ig) E binding patterns to the peanut allergen Ara h 9 and two homologous LTPs (walnut Jug r 3 and peach Pru p 3) between the US and Spain contribute to differences observed in allergic reactivity. Synthetic overlapping 15-amino acid-long peptides offset by five amino acids from Ara h 9, Jug r 3, and Pru p 3 were synthesized, and the intact proteins were attached to microarray slides. Sera from 55 peanut-allergic individuals from the US were tested for IgE binding to the linear peptides and IgE binding to intact proteins using immunofluorescence. For comparison, sera from 17 peanut-allergic individuals from Spain were also tested. Similar IgE binding profiles for Ara h 9, Jug r 3, and Pru p 3 were identified between the US and Spain, with slight differences. Certain regions of the proteins, specifically helices 1 and 2 and the C-terminal coil, were recognized by the majority of the sera more often than other regions of the proteins. While serum IgE from peanut-allergic individuals in the US binds to peptides of Ara h 9 and its homologs, only IgE from the Spanish subjects bound to the intact LTPs. This study identifies Ara h 9, Jug r 3, and Pru p 3 linear epitopes that were previously unidentified using sera from peanut-allergic individuals from the US and Spain. Certain regions of the LTPs are recognized more often in US subjects, indicating that they represent conserved and possible cross-reactive regions. The location of the epitopes in 3D structure models of the LTPs may predict the location of potential conformational epitopes bound by a majority of the Spanish patient sera. These findings are potentially important for development of peptide or protein-targeting diagnostic and therapeutic tools for food allergy.

## Introduction

Food allergies have been rising for the past century and continue to rise worldwide (1). In the Mediterranean area, as well as other European countries, plant food allergy is often attributed to non-specific lipid transfer proteins (LTPs) that can cause a variety of symptoms ranging from mild to severe, depending on co-factor involvement (2–4). These small proteins (∼9 kDa) were first associated with human allergy when two immunoglobulin (Ig) E binding components in apple and peach were identified (5–7). Presently, there are 52 LTP allergens registered in the World Health Organization/International Union of Immunological Societies allergen database (http://allergen.org/), including the peanut LTP Ara h 9 and homologs walnut Jug r 3 and peach Pru p 3.

LTPs are ubiquitous among seed plants and are believed to be involved in plant defense mechanisms against bacterial and fungal infections (4, 8). LTPs have a conserved 3D structure consisting of a hydrophobic, lipid-binding cavity composed of four *α*-helices connected by short loops and stabilized by four disulfide bridges, as seen in the crystal structure of Pru p 3 (PDB ID: 2ALG or 2B5S) (9). The stabilized structure provides protease and thermal resistance to the proteins, thus contributing to their allergenicity (10–12). There have been many studies focused on IgE cross-reactivity of LTPs from botanically related sources (13, 14) and unrelated sources (11, 15–18). High sequence and structural similarities amongst LTPs are the major reason for high IgE cross-reactivity among these proteins (2, 8, 11).

Sensitization to LTPs is strongly associated with geographical location and largely thought to depend on differences in eating habits (2, 8). LTPs from peanut and fruits of the Rosaceae family, most commonly peach, are considered major allergens in the Mediterranean area and often cause oral allergy syndrome (3, 5, 8, 19, 20). There are a few studies from other geographical regions showing reactivity to LTPs, but at a low prevalence in comparison to the Southern Europe and Mediterranean areas (3, 21–24). In regions such as the United States, LTPs are considered minor allergens (3, 22), thus LTP studies in the USA are lacking. The goal of this study is to determine if differences in IgE binding patterns to LTPs between the USA and Spain contribute to differences observed in allergic reactivity. Peptide and whole protein microarray technologies were used with peanut-(*Arachis hypogaea*) allergic individuals' sera to epitope map Ara h 9 as well as two homologous LTPs, Jug r 3 (walnut, *Juglans regia*) and Pru p 3 (peach, *Prunus persica*). The variability in clinical symptoms and geographic distribution of LTP syndrome in Europe makes it a unique type of IgE mediated food allergy. From a diagnostic and therapeutic perspective, the fact that most or all patients are mono-sensitized to a single protein allows for a unique study opportunity, in which the specific location and target of the IgE on that protein can be assessed for clinical relevance. Here, it appears that all three LTPs had similar linear epitope IgE binding profiles, with some differences between the two populations studied (USA and Spain). However, whole protein arrays binding indicates that only IgE from Spanish sera, with known clinical symptoms to LTP were able to bind the intact LTPs.

## Materials and methods

### Sera and microarrays

All sera samples were collected after informed consent and with institutional review board approval. Sera samples were previously collected from 55 US subjects with diagnosed peanut allergy as confirmed by clinical history and oral food challenges. Thirty-three of the US samples were selected from a repository of samples that were collected during the peanut allergy oral immunology clinical studies funded by Aimmune Therapeutics, ARC001(25) (NCT01987817) and PALISADE (26) (ARC003, NCT02635776). US subjects' characteristics are summarized in Table 1. Seventeen sera samples were collected from Spanish subjects with diagnosed peanut allergy as confirmed by clinical history, oral food challenges, skin prick tests, and IgE assessment tests (CAP-RAST). Spanish subjects' characteristics are summarized in Table 2.

**Table 1:** Patient details for USA.

	[ALL] *N* = 55	*N*
Age (yr)	15 [12; 25]	49
Gender:		53
Female	25 (47.2%)	
Male	28 (52.8%)	
Total IgE (IU/ml)	488 [184; 1022]	37
Peanut specific IgE (kUA/L)	68.2 [18.1; 152.9]	38
Ara h 9 specific IgE (kUA/L)	0.35 [0.35; 0.35]	26
Peanut	55 (100%)	55
Walnut	13 (59.1%)^a^	22
Cashew	14 (63.6%)^a^	22
Almond	12 (54.5%)^a^	22
Hazelnut	7 (31.8%)^a^	22
Pecan	12 (54.5%)^a^	22
Pistachio	5 (22.7%)^a^	22

Values are represented as: median [25th %tile; 75th %tile] or *n* (%).

^a^ Percentages were created using the 22 patients with recorded food allergy data.

**Table 2:** Patient details for Spain.

	[ALL] *N* = 17	*N*
Age (yr)	35.5 [28.8; 37.2]	16
Gender:		17
Female	9 (52.9%)	
Male	8 (47.1%)	
Peanut specific IgE (kUA/l)	1.3 [0.7; 2.9]	12
Ara h 9 specific IgE (kUA/l)	0.51 [0; 3.52]	13
Peanut	17 (100%)	17
Walnut	4 (23.5%)	17
Hazelnut	4 (23.5%)	17

Values are represented as: median [25th %tile; 75th %tile] or *n* (%).

Synthetic overlapping 15 amino acid peptides offset by five amino acids, which represent the entire amino acid sequence of allergenic proteins, including the three LTP allergens Ara h 9, Jug r 3, and Pru p 3, were commercially synthesized and spotted onto microarrays slides by JPT Peptide Technologies (Berlin, Germany). Each peptide is represented in triplicate. See Table 3 for LTP allergen details.

**Table 3:** Non-specific LTPs analyzed in this study.

WHO/IUIS name	Allergen isoform	Source	MW (kDa)	AA length	UniProt	GenBank Protein	Published IgE epitopes
Ara h 9	Ara h 9.0201	*Arachis hypogaea* (peanut)	9.1	92	B6CG41	ABX75045	N/A
Jug r 3^a^	Jug r 3.0101	*Juglans regia* (walnut)	11.8	119	C5H617	ACI47547	N/A
Pru p 3^a^	Pru p 3.03	*Prunus persica* (peach)	9.1	91	B6CQU7	ACE80969.1	Pru p 3.0102 11-25, 31-45, 71-80 [1]

^a^ Residues 1-25 of published sequence are not included on array (signal peptide).

Slides were placed in the individual chambers of an HS400 Pro TM (Tecan, San Jose, CA), where they were blocked in 200 µl (all injections were 200 µl) filtered SuperBlock (Thermo-Fisher, Waltham, MA) for 30 min, at room temperature (RT), under agitation. They were then washed for 2 min with Tris-buffered saline containing Tween-20 (100 mM Tris, 274 mM NaCl, 5.4 mM KCl, and 0.5% Tween-20). Subjects' sera were injected into the individual chambers containing the slides and incubated at 4°C overnight (∼16 h) with agitation. Slides were then washed as above before injecting with mouse anti-human IgE (Life Technologies, Grand Island, NY) diluted in SuperBlock (3.3 µg/ml) and incubated for 30 min at RT, washed and incubated with diluted Cy3-conjugated goat anti-mouse IgG (0.4 µg/ml; Life Technologies) for 30 min at RT. After washing and drying, the slides were scanned with a GenePix-4000B scanner (Software: GenePix Pro 7; Molecular Devices, San Jose, CA). IgE binding to the linear peptides was measured by the Cy3 green fluorescence at 532 nm.

### In silico analyses

Peptide fluorescence signal-to-noise ratios (SNRs), as measured by the GenePix Pro 7 software, were summarized by taking the median of available replicate spots. Positive IgE binding was defined as a median fluorescence SNR of 3 or greater (16). Peptides recognized by at least 50% of the sera were considered major IgE reactive peptides (27). To compare the proportion of subjects with positive binding to a given peptide between regions, Fisher exact tests were used. A false discovery rate adjustment was applied to account for multiple comparisons.

Linear IgE reactive peptides were analyzed using the Peptide Similarity tool of the Structural Database of Allergenic Proteins (SDAP; RRID:SCR_012806) (28) for comparison to known allergenic epitopes and potential cross-reactivity. SDAP provides a property distance value (*PD*) for peptide similarity measured by molecular and physical chemical properties of the amino acids and the whole peptide. A low *PD* value (0–3) indicates significantly high similarity or identity between peptides, with a few conservative amino acid substitutions, and a high *PD* value (>10) indicates the peptides are unrelated. *PD* values between 3 and 10 indicate the peptides have recognizable similarity in physical chemical properties.

The Clustal W alignment tool in the MegAlign software (DNASTAR Lasergene, Madison, WI) was used to calculate the identity and similarity of LTP sequences. LTP allergens were modeled using the SWISS-MODEL Protein Modeling Server [SWISS-MODEL, RRID:SCR_018123, ExPASy web server (29)] and PDB: 2B5S [rPru p 3.0102 (9)] as a model template and further analyzed using the Protean 3D software from DNASTAR Lasergene.

### ISAC arrays

Sera samples were also utilized in the detection of IgE binding to intact allergens using ImmunoCAP™ Immuno-Solid phase Allergy Chip (ISAC) 112 specific IgE (sIgE) immunoarrays according to the manufacturer's standard operating procedures and reagents (Thermo-Fisher, Upsala, Sweden). Briefly, ISAC slides containing Ara h 9, Jug r 3, and Pru p 3 were placed in a removable glass slide rack and washed with washing solution for 10 min with vigorous stirring. The slides were then washed with dH_2_O and allowed to dry. The slides were incubated with 30 µl of subjects' serum for each reaction site for 2 h at RT, then washed, dried, and incubated with 30 µl of fluorescence conjugated anti-human IgE antibodies for 30 min. The slides were then washed, dried, and scanned with a LuxScan 10 K Microarray Scanner v. 4.0 (CapitalBio Corp., Beijing, China). The scanned array images were then analyzed using the Phadia Microarray Image Analysis software v. 1.2 to generate IgE signal intensity levels in ISAC Standardized Units (ISU-E) with an operating range of 0.3–100 ISU-E. Wilcoxon rank-sum tests were used to compare IgE levels between geographical regions for each allergen. Positive binding was defined as an ISAC value above 0.3 ISU-E.

## Results

### IgE binding to linear peptides of Ara h 9

While LTPs are fairly well characterized in European countries (20), related studies are lacking in the United States. To gain a better understanding of LTP sensitization in US subjects, IgE binding to linear peptides of the peanut LTP Ara h 9 and 2 homologs was detected in 55 peanut-allergic US sera using microarrays and compared with 17 peanut-allergic Spanish sera (see Tables 1, 2 for subject details).

**Figure 1:**
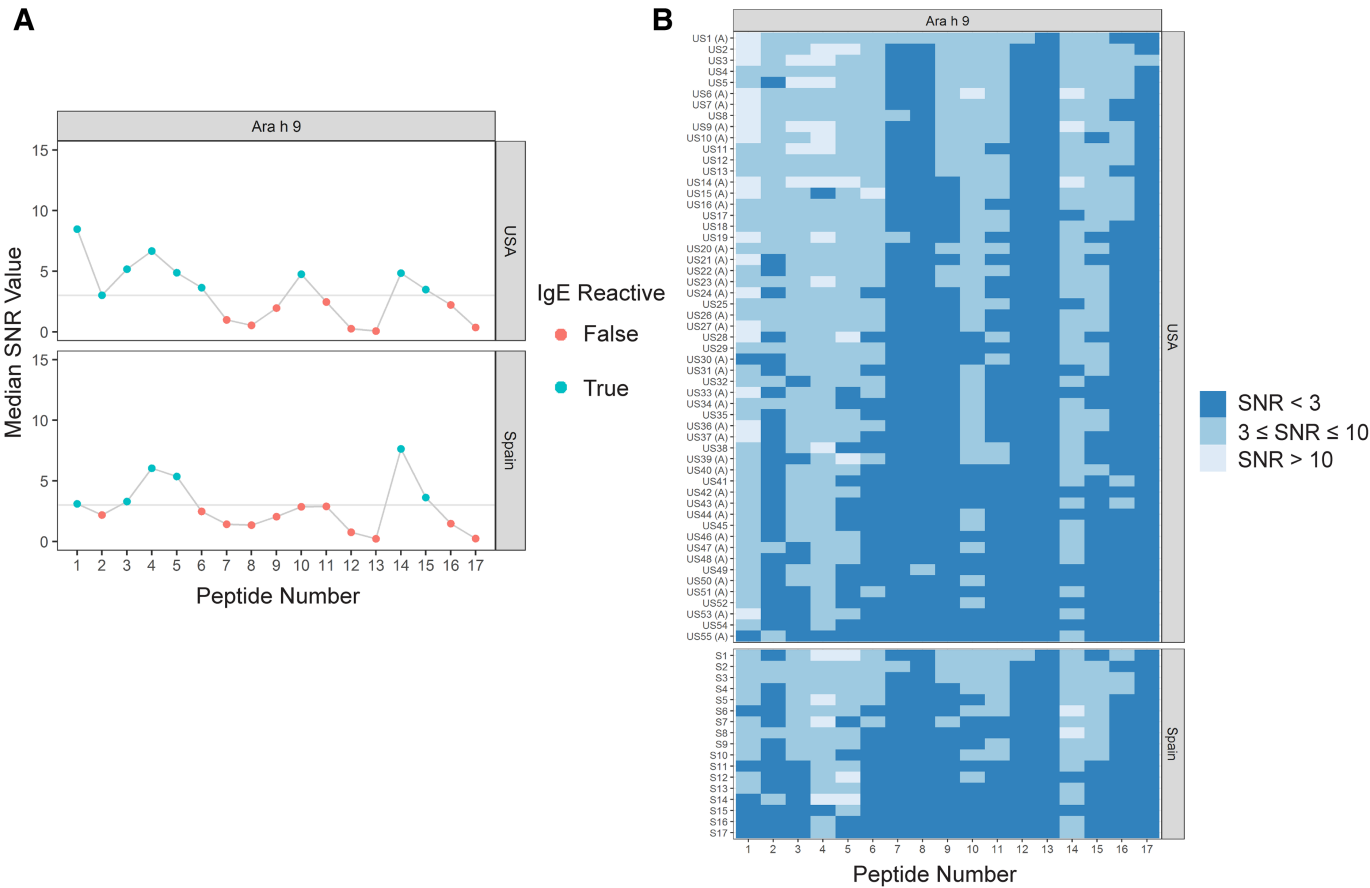
IgE binding to Ara h 9 by peptide and region (USA vs. Spain). (**A**) Dot plots representing the median SNR values for each peptide. Dots are colored blue (True) if the peptide is a major IgE reactive peptide (≥50% of the subjects have positive IgE binding) and pink (False) otherwise. The horizontal line represents the definition for positive binding: SNR ≥ 3. (**B**) Heat maps representing the subject-specific IgE intensities, which are categorized and displayed as follows: SNR < 3 (dark blue), SNR ≥ 3 and ≤10 (medium blue), SNR > 10 (light blue). US samples depicted with (**A**) represent sera samples provided by Aimmune Therapeutics.

Sera IgE taken from US subjects was revealed to have nine major IgE reactive peptides for Ara h 9, including peptides 1–6, 10, 14, and 15 (Figure 1 and Table 4). Peptides 1 and 4 had the highest median IgE binding fluorescence intensity values (median SNR values of 8.5 and 6.7, respectively; Figure 1), with positive sera IgE binding in 96.4% of subjects (Table 5). Collectively, IgE binding covered residues 1–40, 46–60, and 66–85 of the Ara h 9 protein sequence.

**Table 4:** Major IgE binding peptides of Ara h 9 by region.

Peptide #	Sequence	AA range	Sera origin
1	LSCGQVNSALAPCIT	1–15	USA & Spain
2	VNSALAPCITFLTKG	6–20	USA
3	APCITFLTKGGVPSG	11–25	USA & Spain
4	FLTKGGVPSGPCCSG	16–30	USA & Spain
5	GVPSGPCCSGVRGLL	21–35	USA & Spain
6	PCCSGVRGLLGAAKT	26–40	USA
10	AACNCLKAAAGSLHG	46–60	USA
14	AAALPGRCGVSIPYK	66–80	USA & Spain
15	GRCGVSIPYKISTST	71–85	USA & Spain

**Table 5:** Proportion of subjects with positive IgE binding to LTPs by peptide and region.

Protein	Peptide #	Sequence	USA, *n* (%)^a^	Spain, *n* (%)^a^	Adjusted *p*-value^b^
Ara h 9	1	LSCGQVNSALAPCIT	53 (96.4%)	11 (64.7%)	**0.03**
	2	VNSALAPCITFLTKG	28 (50.9%)	4 (23.5%)	0.24
	3	APCITFLTKGGVPSG	46 (83.6%)	10 (58.8%)	0.24
	4	FLTKGGVPSGPCCSG	53 (96.4%)	16 (94.1%)	>0.99
	5	GVPSGPCCSGVRGLL	42 (76.4%)	13 (76.5%)	>0.99
	6	PCCSGVRGLLGAAKT	33 (60%)	6 (35.3%)	0.33
	7	VRGLLGAAKTTADRQ	3 (5.5%)	1 (5.9%)	>0.99
	8	GAAKTTADRQAACNC	2 (3.6%)	0 (0%)	>0.99
	9	TADRQAACNCLKAAA	16 (29.1%)	4 (23.5%)	>0.99
	10	AACNCLKAAAGSLHG	41 (74.5%)	7 (41.2%)	0.15
	11	LKAAAGSLHGLNQGN	23 (41.8%)	8 (47.1%)	>0.99
	12	GSLHGLNQGNAAALP	1 (1.8%)	1 (5.9%)	>0.99
	13	LNQGNAAALPGRCGV	0 (0%)	0 (0%)	>0.99
	14	AAALPGRCGVSIPYK	45 (81.8%)	15 (88.2%)	>0.99
	15	GRCGVSIPYKISTST	30 (54.5%)	9 (52.9%)	>0.99
	16	SIPYKISTSTNCATI	15 (27.3%)	3 (17.6%)	>0.99
	17	ISTSTNCATIKF	1 (1.8%)	0 (0%)	>0.99
Jug r 3	1	AVITCGQVASSVGSC	37 (67.3%)	4 (23.5%)	**0.02**
	2	GQVASSVGSCIGYLR	0 (0%)	2 (11.8%)	0.18
	3	SVGSCIGYLRGTVPT	31 (56.4%)	4 (23.5%)	0.11
	4	IGYLRGTVPTVPPSC	55 (100%)	14 (82.4%)	0.06
	5	GTVPTVPPSCCNGVK	19 (34.5%)	7 (41.2%)	0.94
	6	VPPSCCNGVKSLNKA	36 (65.5%)	13 (76.5%)	0.79
	7	CNGVKSLNKAAATTA	32 (58.2%)	10 (58.8%)	>0.99
	8	SLNKAAATTADRQAA	1 (1.8%)	0 (0%)	>0.99
	9	AATTADRQAACECLK	26 (47.3%)	17 (100%)	**<0.001**
	10	DRQAACECLKKTSGS	21 (38.2%)	3 (17.6%)	0.40
	11	CECLKKTSGSIPGLN	17 (30.9%)	8 (47.1%)	0.43
	12	KTSGSIPGLNPGLAA	2 (3.6%)	2 (11.8%)	0.43
	13	IPGLNPGLAAGLPGK	12 (21.8%)	5 (29.4%)	0.79
	14	PGLAAGLPGKCGVSV	14 (25.5%)	3 (17.6%)	0.94
	15	GLPGKCGVSVPYKIS	55 (100%)	17 (100%)	>0.99
	16	CGVSVPYKISTSTNC	55 (100%)	16 (94.1%)	0.43
	17	PYKISTSTNCKAVK	31 (56.4%)	13 (76.5%)	0.40
Pru p 3	1	LTCPQIQAGLAPCLG	21 (38.2%)	0 (0%)	**0.01**
	2	IQAGLAPCLGYLQRG	38 (69.1%)	4 (23.5%)	**0.01**
	3	APCLGYLQRGGVPAG	22 (40%)	7 (41.2%)	>0.99
	4	YLQRGGVPAGGCCPG	3 (5.5%)	4 (23.5%)	0.21
	5	GVPAGGCCPGIKRLV	54 (98.2%)	13 (76.5%)	0.06
	6	GCCPGIKRLVGSATT	8 (14.5%)	0 (0%)	0.63
	7	IKRLVGSATTTADRQ	2 (3.6%)	1 (5.9%)	0.87
	8	GSATTTADRQNACKC	0 (0%)	0 (0%)	>0.99
	9	TADRQNACKCLKTVA	4 (7.3%)	3 (17.6%)	0.69
	10	NACKCLKTVAGAVKG	10 (18.2%)	2 (11.8%)	>0.99
	11	LKTVAGAVKGINPGY	2 (3.6%)	0 (0%)	>0.99
	12	GAVKGINPGYAAALP	4 (7.3%)	1 (5.9%)	>0.99
	13	INPGYAAALPSLCGV	14 (25.5%)	2 (11.8%)	0.69
	14	AAALPSLCGVKIPYK	54 (98.2%)	16 (94.1%)	0.71
	15	SLCGVKIPYKISAST	40 (72.7%)	10 (58.8%)	0.69
	16	KIPYKISASTNCNSV	22 (40%)	4 (23.5%)	0.69
	17	ISASTNCNSVK	0 (0%)	0 (0%)	>0.99

^a^ Number and percentage of subjects with SNR ≥ 3.

^b^ Fisher exact test *p*-value with FDR adjustment. Bolded values indicate statistical significance.

The major IgE reactive peptides for Ara h 9 identified using sera from Spain were similar to the peptides identified from the US. However, only six of the nine peptides were recognized by the majority of the Spanish subjects (Figure 1 and Table 4). Peptides 2, 6, and 10 had fewer than 50% of subjects with positive binding (SNR ≥ 3), thus were not classified as major IgE reactive peptides in Spain. However, the percentage of subjects with IgE specific to those peptides was not significantly different between the two countries (Table 5). A significantly different percentage of US subjects had positive IgE binding to peptide 1 than Spanish subjects, even though it is considered a major IgE reactive peptide in both populations (96.4% vs. 64.7%, respectively, *p* = 0.03; Table 5). Peptides 4 and 14 had the highest median IgE binding fluorescence intensity values in Spain (median SNR values of 6.0 and 7.6, respectively; Figure 1), with positive sera IgE binding in over 94% and 88% of subjects, respectively (Table 5). Collectively, Spanish sera IgE binding covered residues 1–35 and 66–85 of the Ara h 9 protein sequence.

### Ara h 9 peptide similarity analyses

The nine major IgE reactive peptides for Ara h 9 were analyzed by the Peptide Similarity tool in the Structural Database of Allergenic Proteins (SDAP) Web server for molecular and physical chemical property similarity comparisons to allergenic proteins in the database (28). All nine peptides for Ara h 9 were highly similar to other LTP peptides from many plant sources as seen in the example SDAP results for peptide 1 of Ara h 9 in Table 6. Other non-LTP allergens with a peptide match to Ara h 9 included trypsin and α-amylase inhibitors, major royal jelly proteins, apyrase, 13S globulins, paramyosins, metalloprotease, beta-1,3-glucanase, and bromelain (data not shown). Specifically, peptides 2, 3, 4, 5, 6, and 10 of Ara h 9 had *PD* values ranging from 3 to greater than 10, indicating the peptide matches had a recognizable similarity in physical chemical properties or were unrelated. Peptides 1, 14, and 15 had many matches with very low *PD* values between 0 and 3, indicating a highly significant similarity to many allergenic LTP peptides in the database (see Table 6). Such low *PD* values indicate a high possibility of cross-reactivity and conserved regions among LTPs, which is expected considering LTPs are known to have high sequence identity (2, 8, 11). In addition, it indicates that these peptides from Ara h 9 may be evolutionarily conserved.

**Table 6:** SDAP analyses for Ara h 9 peptide 1_ LSCGQVNSALAPCIT.

Allergen	Source	Function	*PD* Seq. Similarity Index	Start Res.	Matching region	End Res.
Ara h 9.0201	Peanut	nsLTP	0	1	LSCGQVNSALAPCIT	15
Ara h 9.0101	Peanut	nsLTP	1.55	25	ISCGQVNSALAPCIP	39
Pru p 3	Peach	nsLTP	2.51	1	ITCGQVSSALAPCIP	15
Zea m 14.0101	Maize	nsLTP	2.69	29	ISCGQVASAIAPCIS	43
Pru av 3	Cherry	nsLTP	3.06	27	LTCGQVSSNLAPCIA	41
Mor n 3.0101	Black mulberry	nsLTP	3.2	1	ITCGQVSSSLAPCIN	15
Pru d 3	European plum	nsLTP	3.41	1	ITCGQVSSNLAPCIN	15
Pru ar 3	Apricot	nsLTP	3.47	1	ITCGQVSSSLAPCIG	15
Pru p 3	Peach	nsLTP	3.52	1	ITCGQVSSSLAPCIP	15
Hev b 12	Latex	nsLTP	3.56	25	ITCGQVQSALVPCLS	39
Mal d 3	Apple	nsLTP	3.67	25	ITCGQVTSSLAPCIG	39
Pha v 3.0201	Kidney bean	nsLTP	4.19	27	ISCGQVTSSLASCIP	41
Lyc e 3	Tomato	nsLTP	4.19	25	LSCGEVTSGLAPCLP	39
Vit v 1	Grape	nsLTP	4.25	2	VTCGQVASALSPCID	16
Sin a 3.0101	White Mustard	nsLTP	4.43	2	LSCGTVNSNLAACIG	16
Pha v 3.0101	Kidney bean	nsLTP	4.44	25	MTCGQVQSNLVPCVT	39
Hor v 1	Barley	trypsin/*α*amylase inhibitor	4.92	27	LNCGQVDSKMKPCLT	41
Len c 3.0101	Lentil	nsLTP	5.02	27	ISCGAVTSDLSPCLT	41
Pyr c 3	Pear	nsLTP	5.12	25	ITCSQVSANLAPCIN	39
Fra a 3.0102	Strawberry	nsLTP	5.28	27	ITCGQVASNISPCVT	41
Cit l 3	Lemon	nsLTP	5.3	1	ITCGQVTGSLAPXIP	15
Fra a 3.0101	Strawberry	nsLTP	5.43	27	ITCGQVASNISPCLT	41
Bra o 3.0101	Cabbage	nsLTP	5.49	2	ISCGTVTSNLAPCAV	16
Rub i 3.0101	Red raspberry	nsLTP	5.52	27	ITCGQVTQNVAPCFN	41
Pru du 3.0101	Almond	nsLTP	5.63	31	VSCGQVVNNLTPCIN	45
Fra a 3.0201	Strawberry	nsLTP	5.79	27	ITCGQVASSISPCVN	41
Cit r 3.0101	Tangerine	nsLTP	6.04	1	ITXGQVTGSLAPXIA	15
Tri a 14.0101	Wheat	nsLTP	6.18	1	IDCGHVDSLVRPCLS	15
Cas s 8	Chestnut	nsLTP	6.23	2	ITCTQVSKSLMPCLT	16
Art v 3.0301	Mugwort	nsLTP	6.54	27	LTCSDVSTKISPCLS	41
Art v 3.0101	Mugwort	nsLTP	6.63	2	LTCSDVSNKISPCLS	16
Pla a 3.0101	London plane tree	nsLTP	6.7	28	ITCGTVVTRLTPCLT	42
Art v 3.0202	Mugwort	nsLTP	6.78	26	LTCSDVSNKITPCLN	40
Api g 2	Celery	nsLTP	6.89	28	LTCGQVTGKLGGCLG	42
Jug r 3	Walnut	nsLTP	6.91	2	ITCGQVASSVGSCIG	16

Allergens present on the microarrays and analyzed in this study are shaded yellow.

A low *PD* value (0-3) indicates significantly high similarity or identity between peptides. Higher *PD* values (3-10) indicate the peptides have recognizable similarity in physical chemical properties.

**Figure 2:**
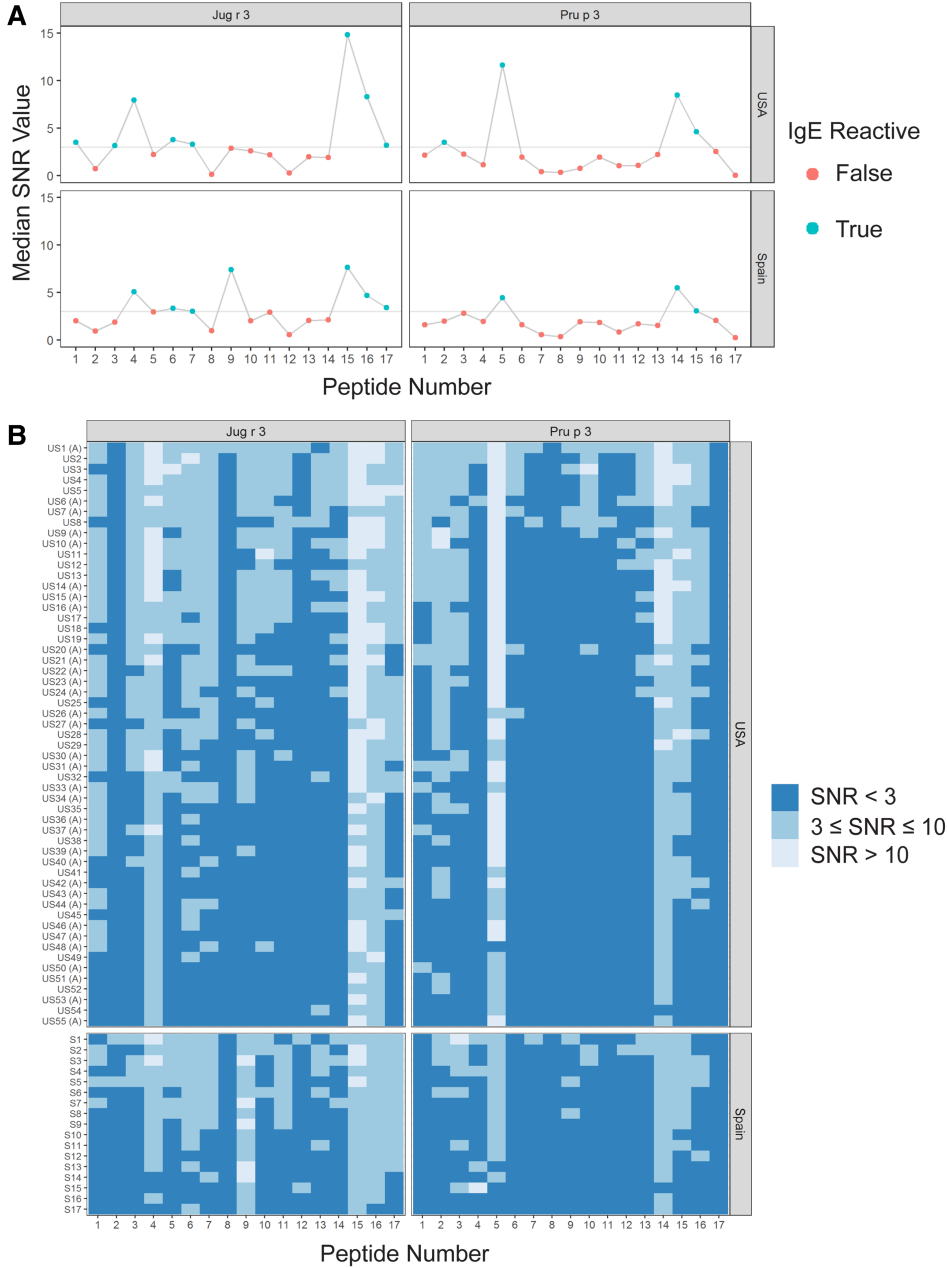
IgE binding to Jug r 3 and Pru p 3 by peptide and region (USA vs. Spain). (**A**) Dot plots representing the median SNR values for each peptide. Dots are colored blue (True) if the peptide is a major IgE reactive peptide (≥50% of the subjects have positive IgE binding) and pink (False) otherwise. The horizontal line represents the definition for positive binding: SNR ≥ 3. (**B**) Heat maps representing the subject-specific IgE intensities, which are categorized and displayed as follows: SNR < 3 (dark blue), SNR ≥3 and ≤10 (medium blue), SNR > 10 (light blue). US samples depicted with (**A**) represent sera samples provided by Aimmune Therapeutics.

**Figure 3:**
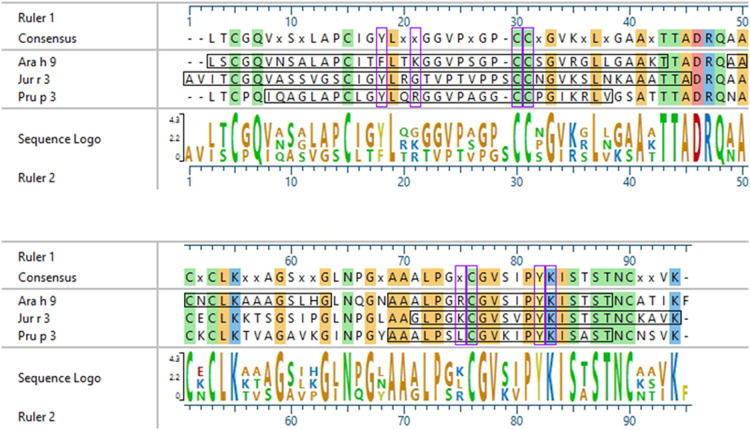
Sequence alignment of Ara h 9, Jug r 3, and Pru p 3. ClustalW (Clustal W2, RRID:SCR_002909) sequence alignment between Ara h 9.0201, Jug r 3.0101, and Pru p 3.03. Consensus sequence is shown above the alignment. Horizontal black boxes indicate IgE binding epitopes from US sera identified in this study. Colored vertical highlights show the conserved amino acids. Amino acids are color coded based on chemistry. Vertical purple boxes indicate basic (K and R), polar (**C**), or aromatic (F and Y) residues within peptides 4, 14, and 15 of Ara h 9 that are conserved or semi-conserved.

Interestingly, there were two commonly known and well-studied LTPs listed in the SDAP results that were also present on our microarray chips, including Jug r 3 from walnut and Pru p 3 from peach (Table 6). Ara h 9 is highly similar to these proteins (76.3% and 76.9% similar to Jug r 3 and Pru p 3, respectively), with identities above 59% (Table 7). To determine if the IgE reactive peptides for Ara h 9 were similar to the homologous LTPs from walnut and peach, IgE binding to the linear peptides of Jug r 3.0101 and Pru p 3.03 were detected using US sera. Jug r 3 had eight major IgE reactive peptides covering residues 1–45 and 71–94, and Pru p 3 had four reactive peptides covering residues 6–35 and 66–85 (Figure 2 and Table 8). Peptides 4, 15, and 16 of Jug r 3 and peptides 5 and 14 of Pru p 3 had the highest median IgE binding fluorescence intensity values relative to other peptides in their respective proteins (all medians greater than 8; Figure 2). Most of the IgE reactive peptides for Ara h 9 were highly similar to the IgE reactive peptides in Jug r 3 and Pru p 3, as seen in the sequence alignment in Figure 3. For example, peptide 4 of Ara h 9 is homologous to peptide 4 of Jug r 3 and peptide 5 of Pru p 3. Also, Ara h 9 peptides 14 and 15 were homologous to peptides 15 and 16 of Jug r 3 and to peptides 14 and 15 of Pru p 3 (Figure 3). This sequence homology indicates possible conserved regions and cross-reactivity among these three proteins, as well as other LTPs.

**Table 7:** Non-specific LTP sequence identity and similarity.

	Ara h 9	Jug r 3	Pru p 3
Ara h 9	100 (100)	61.3 (76.3)	59.3 (76.9)
Jug r 3		100 (100)	54.8 (69.9)
Pru p 3			100 (100)

Values are represented as % identity (% similarity) generated using ClustalW.

**Table 8:** Major IgE binding peptides of Jug r 3 and Pru p 3 by region.

Protein	Peptide #	Sequence	AA range	Sera origin
Jug r 3	1	AVITCGQVASSVGSC	1–15	USA
	3	SVGSCIGYLRGTVPT	11–25	USA
	4	IGYLRGTVPTVPPSC	16–30	USA & Spain
	6	VPPSCCNGVKSLNKA	26–40	USA & Spain
	7	CNGVKSLNKAAATTA	31–45	USA & Spain
	9	AATTADRQAACECLK	41–55	Spain
	15	GLPGKCGVSVPYKIS	71–85	USA & Spain
	16	CGVSVPYKISTSTNC	76–90	USA & Spain
	17	PYKISTSTNCKAVK	81–94	USA & Spain
Pru p 3	2	IQAGLAPCLGYLQRG	6–20	USA
	5	GVPAGGCCPGIKRLV	21–35	USA & Spain
	14	AAALPSLCGVKIPYK	66–80	USA & Spain
	15	SLCGVKIPYKISAST	71–85	USA & Spain

IgE binding to Jug r 3 and Pru p 3 were also detected using Spanish sera for comparison. Seven Jug r 3 peptides were considered major IgE binding peptides using Spanish sera, covering residues 16–55 and 71–94 (Figure 2 and Table 8). Peptides 1 and 3 were considered major IgE reactive peptides in the US but not Spain; however, the proportion of subjects with positive IgE binding to peptide 3 was not different between the two regions (*p* = 0.11; Table 5). Peptide 9 was considered a major peptide in Spain (100% of subjects with positive binding) but not the US (47.3% of subjects with positive binding), a statistically significant difference (*p* < 0.001; Table 5). Every US and Spanish subject had IgE binding to peptide 15 of Jug r 3 (Figure 2 and Table 5). Only three of the Pru p 3 peptides were considered major IgE binding peptides in Spain, including peptides 5, 14, and 15, covering residues 21–35 and 66–85 (Figure 2 and Table 8). Peptide 2 was considered a major IgE reactive peptide in the US but not in Spain, with 69.1% and 23.5% of subjects with positive IgE binding, respectively (*p* = 0.01; Table 5). These data suggest there are conserved regions, especially within the beginning and end of the protein sequences, in LTPs with IgE binding in both the US and Spain.

### IgE binding to intact LTPs

**Figure 4:**
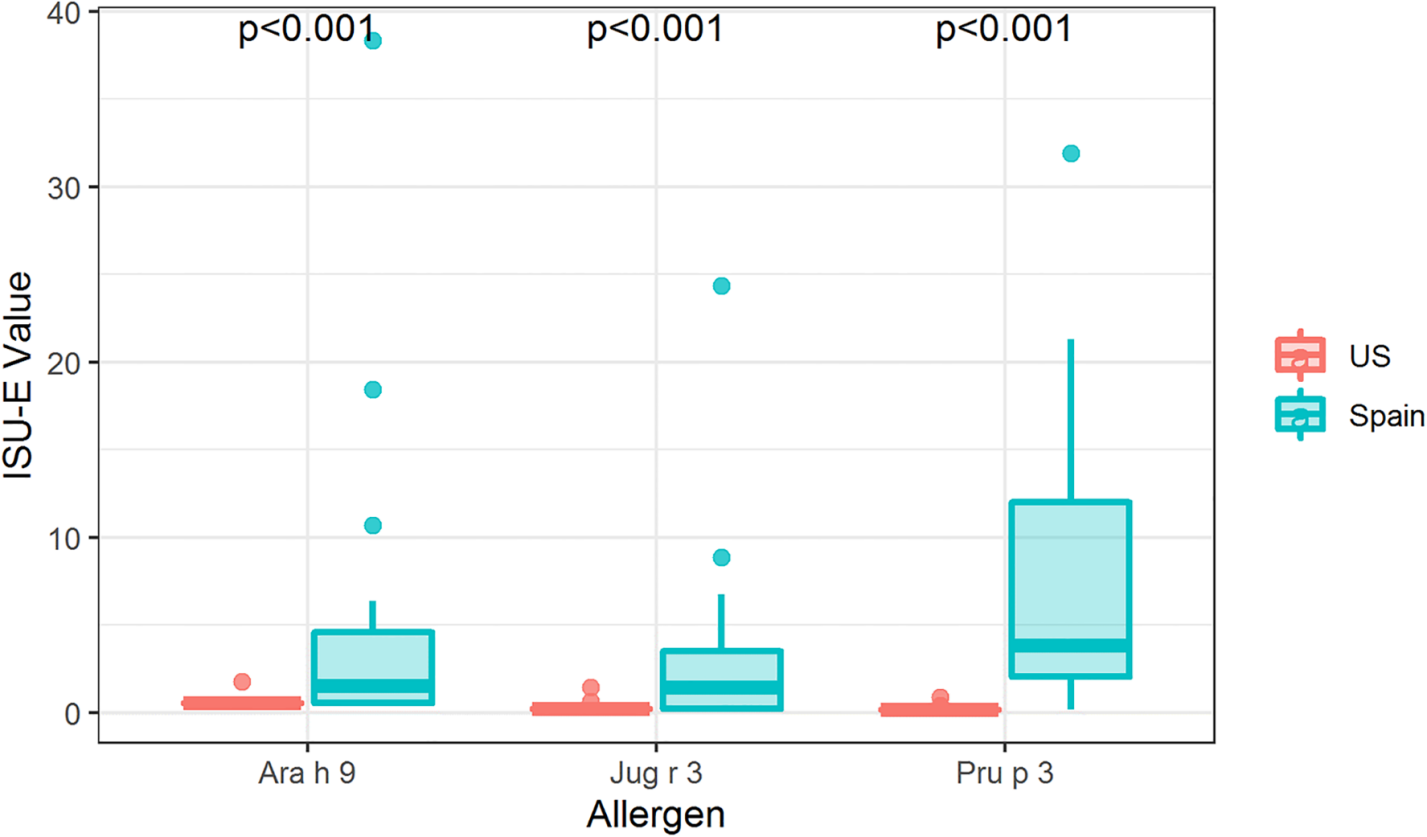
IgE binding to intact LTPs by region. Boxplot depicting ISAC array detection of sera IgE binding to intact Ara h 9, Jug r 3, and Pru p 3 in the US and Spain. *P*-values indicate a significant difference between regions as determined by Wilcoxon rank-sum tests per allergen. US, *N* = 54; Spain, *N* = 17.

To fully understand IgE binding to LTPs, whole protein or conformational IgE binding was considered in addition to identifying the linear IgE reactive peptides. Therefore, IgE binding to intact LTP allergens was detected using ImmunoCAP™ ISAC immunoarrays to determine if there is a conformational aspect to IgE binding to Ara h 9, Jug r 3, and Pru p 3. IgE from US subjects did not bind significantly to the intact proteins Ara h 9, Jug r 3, and Pru p 3 on ISAC arrays (Figure 4), even though IgE binding occurred with the peptide microarrays (Figures 1, 2). Interestingly, most of the Spanish sera had IgE bound to all three intact LTPs, with median ISU-E values statistically higher than US subjects (*p* < 0.001 for all three LTPs; Figure 4). 71% of Spanish sera IgE bound to Ara h 9, 65% bound to Jug r 3, and 76% bound to Pru p 3. It is possible a conformational epitope contributes to IgE binding in Spanish subjects and not US subjects, considering both populations had IgE binding to linear peptides.

**Figure 5:**
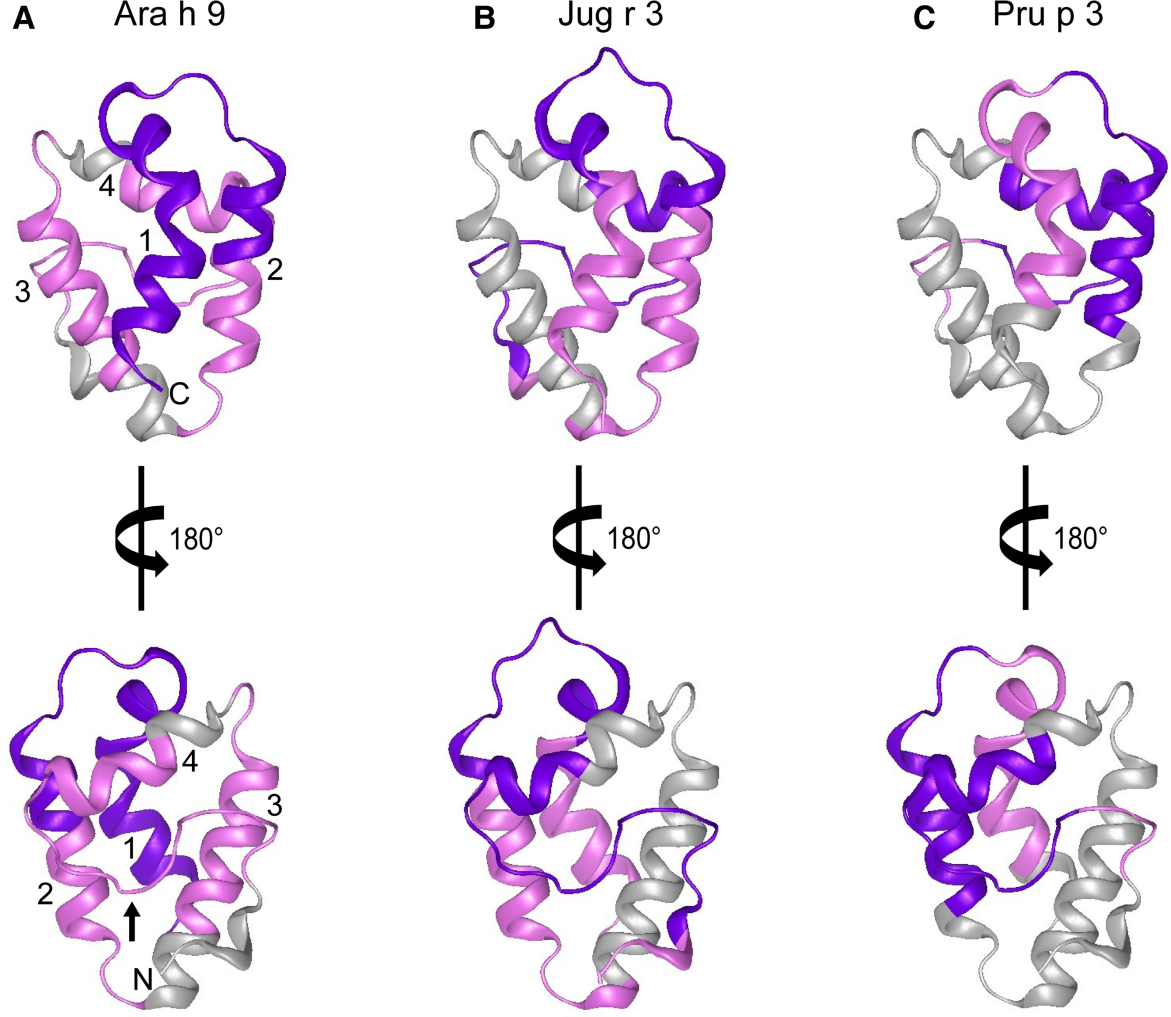
Predicted 3D structures of Ara h 9, Jug r 3, and Pru p 3. The frontal and the 180° rotated views of the predicted 3D structures of (**A**) Ara h 9, (**B**) Jug r 3, and (**C**) Pru p 3 using the SWISS-MODEL Protein Modeling Server (29) and PDB: 2B5S [rPru p 3.0102 (9)] as a model template. N-terminus and C-terminus are indicated in panel A with N and C, respectively. Numbers 1–4 in panel A are representative of the four major α-helices in LTPs. The small black arrow in panel A indicates the C-terminal coil. Major IgE reactive regions identified by US sera are highlighted in pink (median SNR ≥ 3 and <5) and purple (median SNR ≥ 5).

In order to help visualize this possible conformational aspect to the IgE binding to LTPs, modeled 3D structures of Ara h 9, Jug r 3, and Pru p 3 were generated using the SWISS-MODEL Protein Modeling Server (29) and PDB: 2B5S [rPru p 3.0102 (9)] as a model template. The general predicted structure of LTPs consisted of 4 α-helices connected by short loops and a large C-terminal coil (Figure 5), which is consistent with other LTPs crystalized to date (9, 30–36). The major IgE reactive peptides identified from US sera are highlighted on the surface of the structures in Figure 5. IgE binding covered most of the protein surface of Ara h 9, including the entire first two N-terminal helices, most of the third and fourth helices, and the C-terminal coil. Similarly, Jug r 3 and Pru p 3 had IgE binding to helices 1, 2, and 4, and the C-terminal loop (Figure 5). The interhelix loop between helices 1 and 2, which corresponds to GGVPS in Ara h 9, was a conserved IgE binding region among all three LTPs, with median SNR values above 5 (highlighted in purple; Figure 5). The C-terminal coil, found within overlapping peptides 14 and 15 of Ara h 9, was also conserved among all three LTPs as a major IgE binding epitope. These conserved IgE binding regions may cause cross-reactivity among LTPs and are potential conformational epitopes.

## Discussion

This study identifies the linear IgE epitopes for the peanut LTP allergen Ara h 9 as well as homologs Jug r 3 (walnut) and Pru p 3 (peach) with sera taken from peanut-allergic individuals living in the US and Spain. Samples from US subjects showed that the range of IgE binding to Ara h 9 occurred at amino acids 1–40, 46–60, and 66–85, covering the majority of the protein sequence. Similarly, samples from Spanish subjects showed that the IgE binding range covered residues 1–35 and 66–85 of the Ara h 9 protein sequence. IgE epitopes have been previously identified in other LTP allergens, including Tri a 14 (wheat) (37, 38), Pru p 3.0102 (peach) (39), and other Rosaceae fruit LTPs (40), such as Pru ar 3 (apricot), Mal d 3 (apple), and Pru d 3 (plum). However, this is the first time Ara h 9 and Jug r 3, which are considered major allergens in the Mediterranean area (3, 5, 8, 19, 20, 41, 42), have been epitope mapped in the US. The epitopes identified here coincide with the recently identified epitopes of Ara h 9 and Pru p 3 in peach and peanut allergic Spanish individuals (24). In that study, the authors compare the epitopes of a peanut tolerant vs. peanut allergic group and show that the IgG4/IgE ratio of Ara h 9, peptide 4, corresponding to one of the major epitopes identified here (peptides 3–5), was significantly higher in the peanut-tolerant group, with no significant differences in this ratio for the corresponding peptide in Pru p 3, which suggests that IgG4 blocks this major epitope in peanut tolerant individuals (24).

Certain regions of the proteins bind IgE more than other regions, indicating that they represent conserved and possible cross-reactive sequences. For example, IgE binding to peptides 4, 14, and 15 of Ara h 9 are conserved in Jug r 3 and Pru p 3, with both the US and Spanish sera (Figures 1–3) often displaying higher median SNR values. These three LTPs are highly similar to one another (Table 7) as expected (2, 8, 11, 12). It is this similarity that often causes cross-reactivity (35% aa similarity threshold) of conserved IgE binding epitopes between LTPs (11, 13–18, 40). When highlighted on the modeled 3D structures, the conserved regions with IgE reactivity (particularly peptides 4, 14, and 15 of Ara h 9) coincide with the interhelix loop between helices 1 and 2 and the C-terminal coil (Figure 5). These regions in particular, contain conserved positively charged residues (Arg and Lys), the typical Cys residues in LTPs involved in disulfide bridges, and aromatic residues (Phe and Try; purple boxes in Figure 3). Such residues have been shown to be involved with IgE binding and epitope formation in previous studies ((39, 40, 43, 44). It is likely these regions on the proteins are conserved and cross-reactive among LTPs and can indicate potential conformational epitopes.

**Figure 6:**
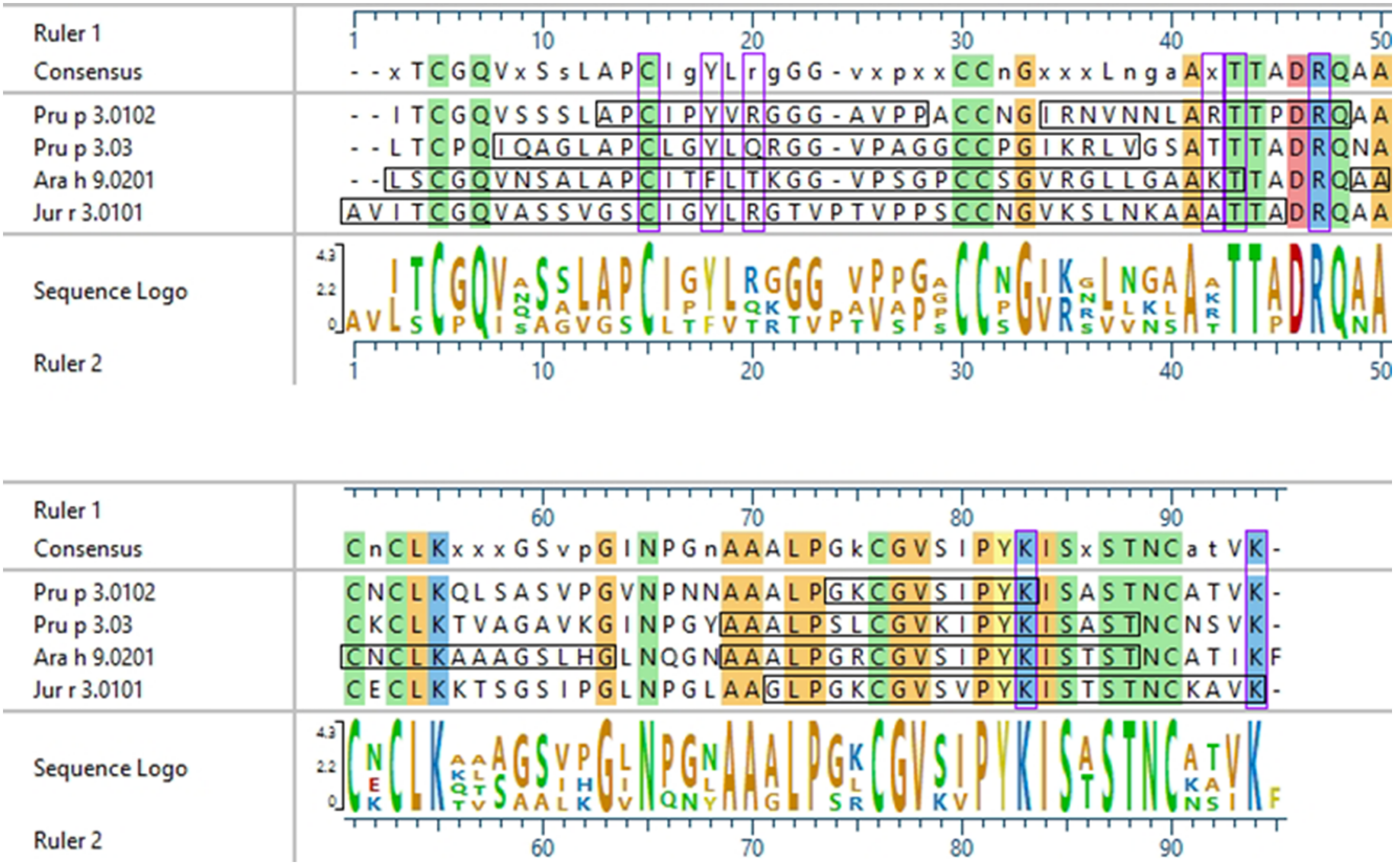
Sequence alignment of Pru p 3.0102 with LTPs from this study. ClustalW sequence alignment between Pru p 3.0102, Pru p 3.03, Ara h 9.0201, and Jug r 3.0101. Consensus sequence is shown above the alignment. Amino acids are color coded based on chemistry, with conserved residues being highlighted vertically. Horizontal black boxes indicate the major IgE binding epitopes identified in this study with US sera or in a previous study for Pru p 3.0102. Vertical purple boxes indicate the specific residues previously predicted to have IgE binding capabilities.

In 2003, García-Casado et al. identified the IgE epitopes of the peach LTP Pru p 3.0102, also highly allergenic in the Mediterranean area (4, 5), using Dot-Blot analyses, and found three major IgE epitopes at residues 11–25, 31–45, and 71–80 (39). Pru p 3.03 analyzed here is 54.9% identical to Pru p 3.0102, and the IgE epitopes defined in García-Casado et al. align to the IgE reactive peptides of Pru p 3.03 defined by our microarray results (Figure 6). Specifically, epitope 1 of Pru p 3.0102 (aa 11–25, APCIPYVRGGGAVPP) partially aligns with peptides 2 and 5 of Pru p 3.03, and epitope 3 of Pru p 3.0102 (aa 71–80, GKCGVSIPYK) is highly similar to peptides 14 and 15 of Pru p 3.03 (Figure 6). These epitopes are further conserved in Ara h 9 and Jug r 3. As LTPs are highly similar and homologous to one another, it is expected that some similarity would be expected among IgE epitopes, which explains the high levels of cross reactivity seen among LTPs from various plant sources (40, 45). One can presume that the epitopes found in our study are likely the accurate epitopes for this allergen; however, further experimentation is required to confirm the specific residues required for IgE binding to Ara h 9 and homologs in different geographical locations. García-Casado et al. also predicted and tested the specific residues likely responsible for IgE binding based on their electrostatic properties (39). The authors mutated those predicted residues and found that they were necessary for IgE binding. These include five positively charged residues: Arg39, Thr40, Arg44, Lys80, and Lys91. When Pru p 3.0102 is aligned to the three LTPs from this study, those five vital amino acids are conserved (Figure 6), possibly indicating that these residues may also play a role in IgE binding to those LTPs and contribute to IgE cross-reactivity.

In this study, we also analyzed IgE binding to intact LTPs using ISAC arrays (Figure 4) and found that sera IgE from Spanish subjects but not US subjects could bind to folded Ara h 9, Jug r 3, and Pru p 3. Both study populations had peanut-specific IgE but US subjects with peanut IgE had relatively higher observed levels (68.2 kUA/L in USA vs. 1.3 kUA/L in Spain; Tables 1, 2). However, both populations had relatively low median values of Ara h 9 sIgE: US subjects 0.35 kUA/L and Spanish subjects 0.51 kUA/L. Typically, US patients with peanut allergies are thought to be sensitized to the major allergens Ara h 1, Ara h 2/6, and Ara h 3 (44), and not Ara h 9 (22). Individuals most commonly allergic to peach from some European and Mediterranean countries tend to be sensitized to LTPs, with often severe reactions (3, 5, 8, 10, 19, 20). One likely theory for this geographical differentiation is the variation in eating habits or patterns of pollen exposure (8). Due to the higher levels of Pru p 3 IgE than related pollens and the lack of full inhibition of IgE binding to Pru p 3 by pollen LTPs, a recent review suggests that LTP-related allergy may not be a pollen-food syndrome (46). Considering the ISAC results and the sIgE levels within both populations, it is plausible that US patients do not react with intact LTP allergens, possibly indicating a cross-reaction is occurring due to IgE binding to linear peptides of Ara h 9. This may also explain why Ara h 9, and LTP-based food allergy are not as important in the US as they are in the Mediterranean area.

Understanding what makes proteins allergens, and improving diagnostic and prediction tools, requires the understanding of how the immune system interacts with allergens and whether these interactions are specific to a geographic location. This study identifies IgE binding epitopes for Ara h 9, Jug r 3, and Pru p 3 using peanut-allergic sera from subjects from the US and Spain. Comparisons show some small differences between the linear epitope maps of the two populations and among the three LTPs assessed, which may indicate peptide-based treatments may enable targeting multiple foods with cross-reactive molecules at the same time. However, it appears that most of the sera IgE from Spain bind to conformational epitope(s) as well as to the linear epitopes. This is likely to explain why allergic individuals react differently to LTP allergens based on geographical location. This finding implies that it may be possible to distinguish clinically relevant IgE binding as well as developing geographically targeted diagnostics and treatments. Further research is necessary to specify amino acids within the epitopes and certain conformations that are directly involved in IgE binding and reaction severity in an allergic population.

## Data Availability

The original contributions presented in the study are included in the article/Supplementary Material, further inquiries can be directed to the corresponding author/s.

## References

[1] Sampath V Abrams EM Adlou B Akdis C Akdis M Brough HA Food allergy across the globe. J Allergy Clin Immunol. (2021) 148(6):1347–64. 10.1016/j.jaci.2021.10.018 34872649

[2] Salcedo G Sánchez-Monge R Barber D Díaz-Perales A. Plant non-specific lipid transfer proteins: an interface between plant defence and human allergy. Biochim Biophys Acta. (2007) 1771(6):781–91. 10.1016/j.bbalip.2007.01.001 17349819

[3] Lauer I Dueringer N Pokoj S Rehm S Zoccatelli G Reese G The non-specific lipid transfer protein, Ara h 9, is an important allergen in peanut. Clin Exp Allergy. (2009) 39(9):1427–37. 10.1111/j.1365-2222.2009.03312.x 19624524

[4] Skypala IJ Bartra J Ebo DG Antje Faber M Fernández-Rivas M Gomez F The diagnosis and management of allergic reactions in patients sensitized to non-specific lipid transfer proteins. Allergy. (2021) 76(8):2433–46. 10.1111/all.14797 33655502

[5] Pastorello EA Farioli L Pravettoni V Ortolani C Ispano M Monza M The major allergen of peach (Prunus persica) is a lipid transfer protein. J Allergy Clin Immunol. (1999) 103(3):520–6. 10.1016/S0091-6749(99)70480-X 10069889

[6] Lleonart R Cisteró A Carreira J Batista A Moscoso del Prado J. Food allergy: identification of the major IgE-binding component of peach (Prunus persica). Ann Allergy. (1992) 69(2):128–30. PMID: 1380782. 1380782

[7] Pastorello EA Pravettoni V Farioli L Ispano M Fortunato D Monza M Clinical role of a lipid transfer protein that acts as a new apple-specific allergen. J Allergy Clin Immunol. (1999) 104(5):1099–106. 10.1016/S0091-6749(99)70095-3 10550759

[8] Egger M Hauser M Mari A Ferreira F Gadermaier G. The role of lipid transfer proteins in allergic diseases. Curr Allergy Asthma Rep. (2010) 10(5):326–35. 10.1007/s11882-010-0128-9 20582490

[9] Pasquato N Berni R Folli C Folloni S Cianci M Pantano S Crystal structure of peach pru p 3, the prototypic member of the family of plant non-specific lipid transfer protein pan-allergens. J Mol Biol. (2006) 356(3):684–94. 10.1016/j.jmb.2005.11.063 16388823

[10] van Ree R. Clinical importance of non-specific lipid transfer proteins as food allergens. Biochem Soc Trans. (2002) 30(6):910–3. 10.1042/bst0300910 12440944

[11] Asero R Mistrello G Roncarolo D de Vries SC Gautier MF Ciurana CLF Lipid transfer protein: a pan-allergen in plant-derived foods that is highly resistant to pepsin digestion. Int Arch Allergy Appl Immunol. (2001) 124(1–3):67–9. 10.1159/000053671 11306929

[12] Missaoui K Gonzalez-Klein Z Pazos-Castro D Hernandez-Ramirez G Garrido-Arandia M Brini F Plant non-specific lipid transfer proteins: an overview. Plant Physiol Biochem. (2022) 171:115–27. 10.1016/j.plaphy.2021.12.026 34992048

[13] Pastorello EA Ortolani C Farioli L Pravettoni V Ispano M Borga Å Allergenic cross-reactivity among peach, apricot, plum, and cherry in patients with oral allergy syndrome: an in vivo and in vitro study. J Allergy Clin Immunol. (1994) 94(4):699–707. 10.1016/0091-6749(94)90177-5 7930303

[14] García-Sellés FJ Díaz-Perales A Sánchez-Monge R Alcántara M Lombardero M Barber D Patterns of reactivity to lipid transfer proteins of plant foods and artemisia pollen: an in vivo study. Int Arch Allergy Appl Immunol. (2002) 128(2):115–22. 10.1159/000059401 12065911

[15] Asero R Mistrello G Roncarolo D Amato S. Relationship between peach lipid transfer protein specific IgE levels and hypersensitivity to non-Rosaceae vegetable foods in patients allergic to lipid transfer protein. Ann Allergy Asthma Immunol. (2004) 92(2):268–72. 10.1016/S1081-1206(10)61559-1 14989398

[16] Palacín A Gómez-Casado C Rivas LA Aguirre J Tordesillas L Bartra J Graph based study of allergen cross-reactivity of plant lipid transfer proteins (LTPs) using microarray in a multicenter study. PLoS ONE. (2012) 7(12):e50799. 10.1371/journal.pone.0050799 23272072 PMC3522694

[17] Díaz-Perales A Lombardero M Sánchez-Monge R García-Selles FJ Pernas M Fernández-Rivas M Lipid-transfer proteins as potential plant panallergens: cross-reactivity among proteins of Artemisia pollen, Castanea nut and Rosaceae fruits, with different IgE-binding capacities. Clin Exp Allergy. (2000) 30(10):1403–10. 10.1046/j.1365-2222.2000.00909.x 10998016

[18] Asero R Mistrello G Roncarolo D Amato S Caldironi G Barocci F Immunological cross-reactivity between lipid transfer proteins from botanically unrelated plant-derived foods: a clinical study. Allergy. (2002) 57(10):900–6. 10.1034/j.1398-9995.2002.t01-1-23541.x 12269935

[19] Pastorello EA D’Ambrosio FP Pravettoni V Farioli L Giuffrida G Monza M Evidence for a lipid transfer protein as the major allergen of apricot. J Allergy Clin Immunol. (2000) 105(2, Part 1):371–7. 10.1016/S0091-6749(00)90090-3 10669861

[20] Krause S Reese G Randow S Zennaro D Quaratino D Palazzo P Lipid transfer protein (Ara h 9) as a new peanut allergen relevant for a Mediterranean allergic population. J Allergy Clin Immunol. (2009) 124(4):771–8.e5. 10.1016/j.jaci.2009.06.008 19665774

[21] Azofra J Berroa F Gastaminza G Saiz N Gamboa PM Vela C Lipid transfer protein syndrome in a non-mediterranean area. Int Arch Allergy Appl Immunol. (2016) 169(3):181–8. 10.1159/000445893 27144406

[22] Vereda A van Hage M Ahlstedt S Ibañez MD Cuesta-Herranz J van Odijk J Peanut allergy: clinical and immunologic differences among patients from 3 different geographic regions. J Allergy Clin Immunol. (2011) 127(3):603–7. 10.1016/j.jaci.2010.09.010 21093026

[23] Skypala IJ Cecchi L Shamji MH Scala E Till S. Lipid transfer protein allergy in the United Kingdom: characterization and comparison with a matched Italian cohort. Allergy. (2019) 74(7):1340–51. 10.1111/all.13747 30762886 PMC6767535

[24] Sánchez-Ruano L Fernández-Lozano C Ferrer M Gómez F de la Hoz B Martínez-Botas J Differences in linear epitopes of Ara h 9 recognition in peanut allergic and tolerant, Peach Allergic Patients. Front Allergy. (2022) 3:896617. 10.3389/falgy.2022.896617 35935018 PMC9352880

[25] Bird JA Spergel JM Jones SM Rachid R Assa'ad AH Wang J Efficacy and safety of AR101 in oral immunotherapy for peanut allergy: results of ARC001, a randomized, double-blind, placebo-controlled phase 2 clinical trial. Journal Allergy Clin Immunol Pract. (2018) 6(2):476–85.e3. 10.1016/j.jaip.2017.09.016 29092786

[26] Vickery BP Vereda A Casale TB Beyer K du Toit G Hourihane OJ AR101 oral immunotherapy for peanut allergy. N Engl J Med. (2018) 379(21):1991–2001. 10.1056/NEJMoa1812856 30449234

[27] Otsu K Guo R Dreskin SC. Epitope analysis of Ara h 2 and Ara h 6: characteristic patterns of IgE-binding fingerprints among individuals with similar clinical histories. Clin Exp Allergy. (2015) 45(2):471–84. 10.1111/cea.12407 25213872 PMC4470374

[28] Schein CH Ivanciuc O Braun W. Bioinformatics approaches to classifying allergens and predicting cross-reactivity. Immunol Allergy Clin North Am. (2007) 27(1):1–27. 10.1016/j.iac.2006.11.005 17276876 PMC1941676

[29] Waterhouse A Bertoni M Bienert S Studer G Tauriello G Gumienny R SWISS-MODEL: homology modelling of protein structures and complexes. Nucleic Acids Res. (2018) 46(W1):W296–W303. 10.1093/nar/gky427 29788355 PMC6030848

[30] Lee JY Min K Cha H Shin DH Hwang KY Suh SW. Rice non-specific lipid transfer protein: the 1.6 A crystal structure in the unliganded state reveals a small hydrophobic cavity. J Mol Biol. (1998) 276(2):437–48. 10.1006/jmbi.1997.1550 9512714

[31] Charvolin D Douliez JP Marion D Cohen-Addad C Pebay-Peyroula E. The crystal structure of a wheat nonspecific lipid transfer protein (ns-LTP1) complexed with two molecules of phospholipid at 2.1 A resolution. Eur J Biochem. (1999) 264(2):562–8. 10.1046/j.1432-1327.1999.00667.x 10491104

[32] Offermann LR Bublin M Perdue ML Pfeifer S Dubiela P Borowski T Structural and functional characterization of the hazelnut allergen Cor a 8. J Agric Food Chem. (2015) 63(41):9150–8. 10.1021/acs.jafc.5b03534 26417906 PMC4616228

[33] Jain A Salunke DM. Crystal structure of nonspecific lipid transfer protein from Solanum melongena. Proteins. (2017) 85(10):1820–30. 10.1002/prot.25335 28612368

[34] Da Silva P Landon C Industri B Marais A Marion D Ponchet M Solution structure of a tobacco lipid transfer protein exhibiting new biophysical and biological features. Proteins. (2005) 59(2):356–67. 10.1002/prot.20405 15726627

[35] Han GW Lee JY Song HK Chang C Min K Moon J Structural basis of non-specific lipid binding in maize lipid-transfer protein complexes revealed by high-resolution x-ray crystallography. J Mol Biol. (2001) 308(2):263–78. 10.1006/jmbi.2001.4559 11327766

[36] Melnikova DN Mineev KS Finkina EI Arseniev AS Ovchinnikova TV. A novel lipid transfer protein from the dill Anethum graveolens L.: isolation, structure, heterologous expression, and functional characteristics. J Pept Sci. (2016) 22(1):59–66. 10.1002/psc.2840 26680443

[37] Tordesillas L Pacios LF Palacin A Quirce S Armentia A Barber D Molecular basis of allergen cross-reactivity: non-specific lipid transfer proteins from wheat flour and peach fruit as models. Mol Immunol. (2009) 47(2–3):534–40. 10.1016/j.molimm.2009.07.028 19846220

[38] Denery-Papini S Bodinier M Pineau F Triballeau S Tranquet O Adel-Patient K Immunoglobulin-E-binding epitopes of wheat allergens in patients with food allergy to wheat and in mice experimentally sensitized to wheat proteins. Clin Exp Allergy. (2011) 41(10):1478–92. 10.1111/j.1365-2222.2011.03808.x 21771117

[39] García-Casado G Pacios LF Díaz-Perales A Sánchez-Monge R Lombardero M García-Selles FJ Identification of IgE-binding epitopes of the major peach allergen Pru p 3. J Allergy Clin Immunol. (2003) 112(3):599–605. 10.1016/S0091-6749(03)01605-1 13679821

[40] Borges JP Barre A Culerrier R Granier C Didier A Rougé P. Lipid transfer proteins from Rosaceae fruits share consensus epitopes responsible for their IgE-binding cross-reactivity. Biochem Biophys Res Commun. (2008) 365(4):685–90. 10.1016/j.bbrc.2007.11.046 18036340

[41] Dubiela P Del Conte R Cantini F Borowski T Aina R Radauer C Impact of lipid binding on the tertiary structure and allergenic potential of Jug r 3, the non-specific lipid transfer protein from walnut. Sci Rep. (2019) 9(1):2007. 10.1038/s41598-019-38563-1 30765752 PMC6376136

[42] Pastorello EA Farioli L Pravettoni V Robino AM Scibilia J Fortunato D Lipid transfer protein and vicilin are important walnut allergens in patients not allergic to pollen. J Allergy Clin Immunol. (2004) 114(4):908–14. 10.1016/j.jaci.2004.06.020 15480333

[43] Willison LN Zhang Q Su M Teuber SS Sathe SK Roux KH. Conformational epitope mapping of Pru du 6, a major allergen from almond nut. Mol Immunol. (2013) 55(3–4):253–63. 10.1016/j.molimm.2013.02.004 23498967

[44] Palladino C Breiteneder H. Peanut allergens. Mol Immunol. (2018) 100:58–70. 10.1016/j.molimm.2018.04.005 29680589 PMC7060077

[45] Gonzalez-Klein Z Pazos-Castro D Hernandez-Ramirez G Garrido-Arandia M Diaz-Perales A Tome-Amat J. Lipid ligands and allergenic LTPs: redefining the paradigm of the protein-centered vision in allergy. Front Allergy. (2022) 3:864652. 10.3389/falgy.2022.864652 35769581 PMC9234880

[46] Asero R Brusca I Cecchi L Pignatti P Pravettoni V Scala E Why lipid transfer protein allergy is not a pollen-food syndrome: novel data and literature review. Eur Ann Allergy Clin Immunol. (2022) 54(5):198–206. 10.23822/EurAnnACI.1764-1489.206 34092069

